# Posterior deformity correction and internal fixation for sagittal imbalance due to spontaneous fusion in lumbar spondylolisthesis: A case report

**DOI:** 10.1097/MD.0000000000046432

**Published:** 2026-01-02

**Authors:** Yuhai Yan, Changliang Ou, Xu Gao, Haozhu Chen, Kaiwei Zhang

**Affiliations:** a Guizhou University of Traditional Chinese Medicine, Guiyang, Guizhou Province, China; b The First Affiliated Hospital of Guizhou University of Traditional Chinese Medicine, Guiyang, Guizhou Province, China.

**Keywords:** internal fixation, lumbar spondylolisthesis, sagittal imbalance, spontaneous fusion

## Abstract

**Rationale:**

Lumbar spondylolisthesis (LS) with spontaneous fusion (SF) accompanied by sagittal imbalance (SI) poses a practical decision-making challenge in surgical planning. Here, we present a trauma-associated case of lumbar spondylolisthesis with SF and SI, and outline a reproducible, evidence-informed framework for surgical management.

**Patient concerns:**

A 57-year-old patient developed low back pain after trauma. Lumbar computed tomography (CT) showed L4 anterolisthesis with bilateral pars interarticularis defects. Despite standardized conservative treatment, the back pain progressed, with forward trunk inclination of approximately 40° and concomitant left lower-limb weakness.

**Diagnosis:**

Lumbar spondylolisthesis with nerve root impairment.

**Interventions:**

After over 20 years of conservative treatment, the symptoms did not achieve meaningful improvement; the patient’s back pain progressively worsened, with forward trunk inclination of approximately 40° and progressive weakness of the left lower limb. The patient ultimately underwent pedicle screw instrumentation.

**Outcomes:**

The procedure was uneventful; the patient remained on bed rest for 3 days and was discharged on postoperative day 12.

**Lessons:**

LS with SF and SI is encountered clinically but remains under-specified for surgical decision-making; importantly, when LS and SI coexist, symptoms are not invariably attributable to the slipped level – marked forward stooping may instead reflect ligamentum flavum hypertrophy, nerve-root entrapment, adhesions, or other adjacent-level pathology. In selected patients, LS with SI does not require reduction of the slipped vertebra; surgical objectives can be achieved by decompression of the symptomatic level(s) and restoration of segmental lordosis to re-establish global alignment.

## 1. Introduction

LS with spontaneous fusion (SF) and sagittal imbalance (SI) is encountered clinically but remains under-specified for surgical decision-making. It typically reflects intervertebral instability and disruption of sagittal alignment associated with LS. Patients may present with persistent low-back pain, radicular symptoms from nerve-root compression, and gait disturbance.^[1,2]^ SF denotes gradual, nonoperative fusion at the slipped level driven by long-standing instability or local biomechanical changes; paradoxically, SF can coexist with or contribute to SI, further worsening global spinal biomechanics.^[3]^

The pathogenesis remains incompletely understood; however, displacement at the slipped level and the development of SF may alter spinal load-bearing mechanics, precipitating SI. Particularly in older adults, disruption of sagittal alignment can result in substantial neurological impairment and a marked decline in quality of life.^[4,5]^ This report presents a patient with LS with SF and SI who, after prolonged conservative management without meaningful improvement, underwent corrective posterior instrumentation and fusion. Through this case, we discuss clinical diagnostic considerations and surgical strategy selection for this scenario and provide evidence to inform practice.

Publication of this case report and associated images was approved by the patient (and legal guardian, where applicable) through written informed consent. A copy of the signed consent can be provided to the Editor-in-Chief upon request. The authors declare no external funding and no conflicts of interest. All datasets generated and/or analyzed during this study are publicly available (repository/link available upon request). In accordance with local regulations, this single-patient case report did not require review by an institutional review board/local ethics committee. Written informed consent for publication of clinical details and images was obtained from the patient.

## 2. Case presentation

A 57-year-old male of Han ethnicity, employed as a manual worker, presented with trauma-related low-back pain accompanied by left anterolateral lower-limb pain, without intermittent claudication. Symptoms improved with rest but recurred after exertion. During the course of illness, the patient received intermittent conservative management consisting of oral nonsteroidal anti-inflammatory drugs (NSAIDs; e.g., Suo-mi-tong tablets), continuous lumbar traction, and topical anti-inflammatory analgesic patches. Subsequently, low back and left lower-limb pain worsened, with intermittent claudication at approximately 100 m and post-activity exacerbation. Lumbar computed tomography (CT) at a local hospital showed: L5 pars interarticularis defects with grade II anterolisthesis of L5 and L5–S1 fusion; degenerative changes.

On presentation to our hospital, specialty examination revealed a midline spine without obvious kyphosis or scoliosis; intact skin without rash, erythema, ulceration, or sinus tract; local tenderness over L3–S1 spinous processes and paraspinal muscles (+). Range of motion: forward flexion 60°, extension 15°, lateral bending 15°/15°, and axial rotation 15°/15°. Straight-leg raise and reinforcement tests were positive bilaterally; piriformis tension test was borderline (±); prone instability/abdominal bracing test was positive. Neurologically, left lower-limb strength was 5−/5 and right was 5/5; tone and deep tendon reflexes were normal; pathologic signs were absent; superficial sensation of both lower limbs was symmetric; distal perfusion and mobility were preserved, with palpable dorsalis pedis pulses.

Laboratory tests – including complete blood count, coagulation profile, infectious markers, C-reactive protein, and liver/renal function – were unremarkable, with a mildly elevated erythrocyte sedimentation rate.

Imaging: Lumbar digital radiography showed a transitional vertebra, lumbosacral spondylolisthesis with a lumbosacral block vertebra, and degenerative changes. Lumbar CT demonstrated: L3/4 disc bulging with mild protrusion and mild spinal-canal stenosis at L3/4 and L4/5; bilateral L4 pars defects with grade II anterolisthesis and partial L4–L5 fusion; degenerative changes of the lumbar spine and both sacroiliac joints; L1/2 disc degeneration with vacuum phenomenon; suspected sacralization of L5. Lumbar magnetic resonance imaging (MRI) showed: degeneration and bulging at L2/3 and L3/4, with adjacent endplate inflammation at L4/5; grade II anterolisthesis of L4 with obliteration of the L4/5 disc space (clinical correlation advised); lumbar degenerative changes; possible lumbosacral sacralization (clinical correlation advised). Full-length spine digital radiography revealed: a thoracolumbar transitional vertebra – consider thoracization of L1; lumbosacral spondylolisthesis with a lumbosacral block vertebra; degenerative changes and scoliosis of the cervical, thoracic, and lumbar spine.

On comprehensive review, the patient had grade II anterolisthesis of L4 with partial L4 to 5 fusion, L3/4 disc bulging with mild protrusion, and mild canal stenosis at L3/4 and L4/5. Considering symptoms and ancillary findings, compression of the L4 and L5 nerve roots was deemed likely. The patient met surgical indications; after excluding contraindications, we planned posterior L3/4 and L4/5 complete laminectomy and decompression, canal enlargement, nerve-root exploration and adhesiolysis, discectomy, interbody bone-graft fusion, and pedicle-screw fixation.

Operative record:After induction of general anesthesia, the patient was placed prone; the lumbosacral region was prepared and draped in the usual sterile fashion. A midline longitudinal incision of approximately 10 cm centered from L3 to L5 was made. The subcutaneous tissue and superficial/deep fascia were opened in layers; paraspinal muscles were bluntly detached along the lamina to expose the superior and inferior facets of L3, L4, and L5. Paraspinal soft tissue was removed and the bilateral facet joints of L3, L4, and L5 were exposed. Under a standardized technique, pedicle entry points were selected at the junction of the lateral edge of the superior articular process and the base of the transverse process (slightly caudal); axial medialization was approximately 10° to 15°, and the sagittal trajectory was parallel to the superior endplate or with 5° to 10° cranial tilt. For L5, the entry was at the junction just inferolateral to the superior facet and the transverse process base, with greater medialization (approximately 15°–25°) and 10° to 15° cranial tilt. A ball-tipped probe confirmed intact 5 walls; bilateral guidewires were then placed through the L3, L4, and L5 pedicles. Fluoroscopy confirmed satisfactory angles, positions, and depths. Four 6.5 × 50 mm polyaxial pedicle screws were inserted bilaterally at L3 and L4, and 2 6.5 × 45 mm polyaxial screws were inserted bilaterally at L5. C-arm fluoroscopy confirmed satisfactory screw placement.^[6]^ The spinous processes and the supraspinous and interspinous ligaments were removed; bilateral partial laminectomy and partial facetectomy were performed at L3, L4, and L5. Intraoperative exploration revealed L4 bilateral pars defects with L4 anterolisthesis, marked ligamentum flavum hypertrophy, canal stenosis, dural sac compression with poor pulsation, and nerve-root entrapment with adhesions. The exiting and traversing nerve roots were exposed; tortuous epidural veins were noted, and adhesiolysis of the adherent roots was carried out. After protecting the nerves, the lateral recesses were enlarged. On the right side, the L3/4 disc was removed; intervertebral tissue was cleared and the endplates were prepared. The disc space was copiously irrigated with saline. Bilateral rods were implanted, the L3/4 interspace was distracted appropriately, and fixation was achieved. Morselized local autologous cancellous bone (from spinous processes/laminae/facets) was packed for interbody fusion at L3/4 and placed in the bilateral posterolateral gutters until densely filled with firm endplate–bone contact; no allograft or interbody cage was used. Bilateral rods were secured and set screws were tightened. Fusion assessment will follow predefined criteria: dynamic lateral flexion–extension radiographs showing < 3 mm translation or < 3° angular motion, combined with CT evidence of continuous interbody trabeculation and absence of peri-implant radiolucency.^[7]^ The wound was soaked with diluted povidone–iodine, irrigated thoroughly with isotonic saline, and 1 drain was placed. Layered closure of muscle, fascia, subcutaneous tissue, and skin was performed, followed by dressing application. Intraoperative blood loss was approximately 2000 mL; 100 mL of autologous blood was reinfused; 1 U leukocyte-reduced packed red cells and 200 mL fresh frozen plasma were transfused. Indications for autologous and allogeneic transfusion and safety monitoring indices followed classical references.^[8]^ The procedure was completed.

**Figure 1.**
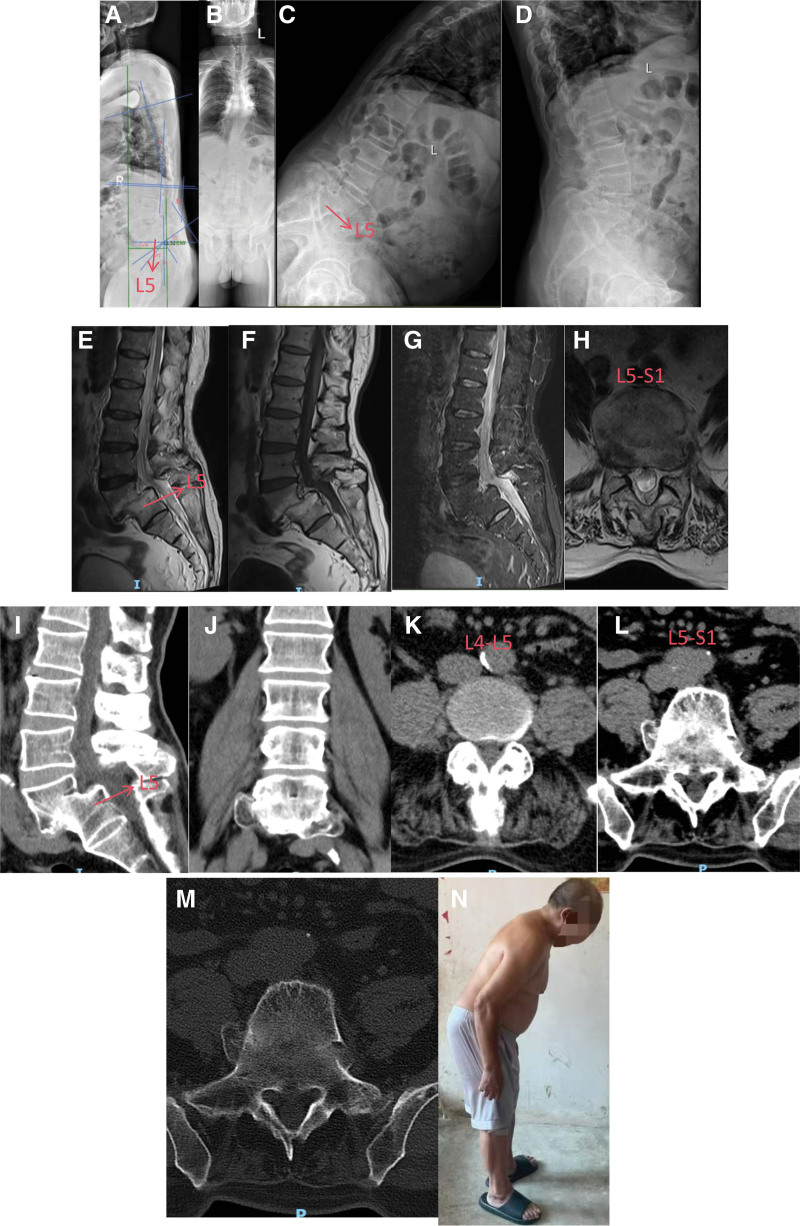
(A and B) Radiographs demonstrate lumbosacral spondylolisthesis with a lumbosacral block vertebra; SVA = 11.52 cm; LL = 39°; TK = 18°; pelvic parameters: PI = 75°; PT = 40°; SS = 35° – indicative of severe sagittal imbalance. (C and D) Flexion–extension lumbar radiographs reveal partial L4 to 5 fusion with stable alignment. (E–H) MRI shows grade II anterolisthesis of L4 with obliteration of the L4 to 5 disc space. (I–M) CT shows: (1) L3–4 disc bulging with mild protrusion and mild canal stenosis at L3–4 and L4 to 5. (2) Bilateral L4 pars interarticularis defects with grade II anterolisthesis and partial L4 to 5 fusion. (N) Demonstrates ~40° anterior trunk inclination in the sagittal plane. (two independent spine surgeons performed blinded, repeated measurements with concordant results).

**Figure 2.**
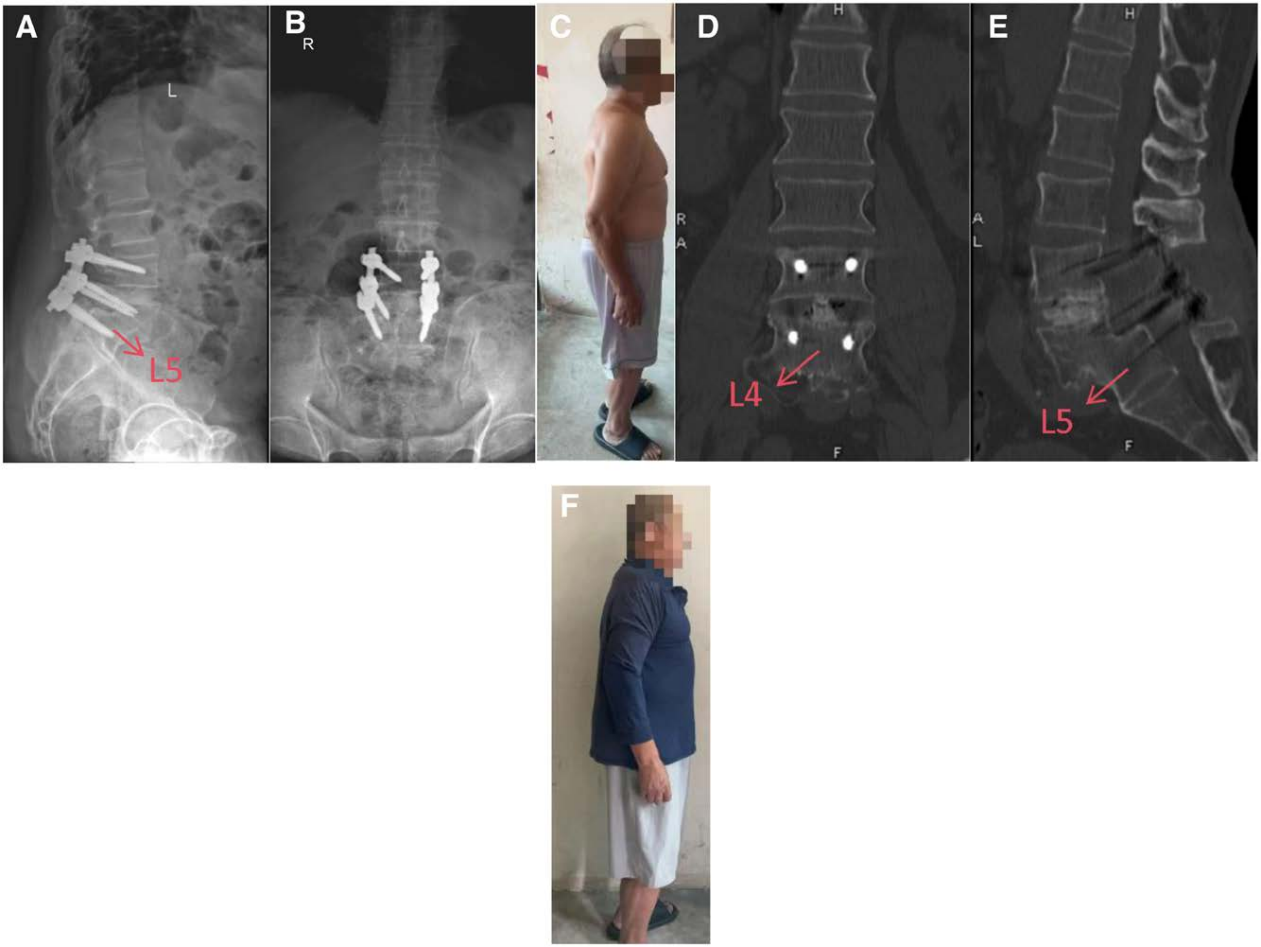
(A–B) Radiographs demonstrate appropriate placement of the screw–rod instrumentation with stable fixation in situ. (C) Shows resolution of the patient’s sagittal anterior trunk inclination, indicating surgical success. (D and E) CT demonstrates early graft incorporation at L3–L4; the pedicle-screw–rod construct is well positioned with fixation maintained in situ. (F) shows the patient’s upper-body forward-lean angle at 0°, indicating a marked short-term postoperative effect.

Intraoperative blood loss of approximately 2000 mL was traced predominantly to an engorged epidural venous plexus and cancellous oozing from the lamina/facet surfaces, associated with extensive adhesiolysis in the setting of hypertrophied ligamentum flavum; no arterial variation or major vascular injury was identified. Hemostasis was achieved with a multimodal approach, including low–central venous pressure positioning/anesthetic management, intermittent gauze packing with pressure, bipolar coagulation, and topical oxidized regenerated cellulose, with staged decompression and repeated irrigation. Given the patient’s advanced age, prolonged operative time, substantial blood loss, and borderline hypotension, he was transferred to the intensive care unit postoperatively. After 3 days of intensive care unit support, he returned to the orthopedic ward. Low-back pain was markedly relieved and there were no residual bilateral lower-limb symptoms. Small intramuscular and intermuscular venous thrombi were detected in both lower limbs; anticoagulation with rivaroxaban was initiated, and follow-up imaging-confirmed resolution. The patient was discharged on postoperative day 12. At 1-month follow-up, bilateral leg pain had resolved, low-back pain was significantly improved, and the sagittal forward inclination improved from ~40° to near-normal. Overall, the surgical treatment was effective and the patient’s quality of life improved substantially. Preoperative examinations and patient status are detailed in Figure 1, with postoperative outcomes at 1 and 3 months provided in Figure 2.

## 3. Discussion

LS with SF and SI is a clinically encountered but decision-making–complex spinal condition. SF typically arises from long-standing spinal instability, leading to gradual bony fusion at the slipped segment; although such natural fusion may relieve some symptoms, it frequently coexists with SI, thereby altering spinal biomechanics.^[2]^ In this case, the patient’s L4 to 5 slip had already undergone SF, yet the resulting SI led to persistent nerve-root compression and worsening clinical symptoms. While deformity correction with internal fixation is often considered, we did not reduce the fused slipped level; instead, we performed decompression and fusion of the adjacent segment to restore sagittal balance and alleviate neural compression.

Although the pathogenesis of LS is relatively well defined – principally linked to spinal instability and excessive loading, Given that the lumbar spine bears greater loads than the cervical and thoracic segments, its clinical significance is particularly pronounced.^[9]^ the specific relationship between SF and SI remains insufficiently elucidated at a mechanistic level. SF may confer segmental stability; however, the combination of vertebral slippage and bony fusion can redistribute spinal loads and thereby provoke changes in sagittal alignment.^[10]^ In this case, the patient had L4 to 5 LS with SF, and pronounced SI exacerbated nerve-root compression, resulting in persistent low-back pain and radicular leg pain that ultimately necessitated surgical intervention. Imaging (radiographs, CT, and MRI) is essential in complex presentations of SI because it provides biomechanical information that directly informs decision-making.^[11]^ Notably, when SF develops, segmental micromotion at the fused level is markedly reduced and that level contributes less to overall lumbar lordosis; in the presence of PI–LL mismatch and an increased SVA (forward trunk shift), global malalignment can become the dominant driver of symptoms.^[12]^ In this patient, preoperative parameters were SVA = 11.52 cm; LL = 39°; TK = 18°; PI = 75°; PT = 40°; SS = 35°, indicating marked SI that worsened nerve-root compression and led to refractory back and leg pain, thereby prompting surgery. Imaging with X-ray, CT, and MRI was therefore pivotal for diagnosing SI and guiding the operative plan.^[11]^

Given bony fusion at the slipped level, forcibly reducing the slipped vertebra may carry unpredictable risks of neural injury and major vascular rupture. Accordingly, the operative strategy focused on targeted nerve-root decompression with fusion of the adjacent levels. We elected to decompress and fuse L3 to 5 to partially correct SI, improve the spine’s biomechanical profile, and relieve neural compression.^[13]^ This strategy effectively addressed the symptomatic level(s) while avoiding the additional risks associated with overcorrection. Postoperatively, the patient’s symptoms improved markedly, indicating that this approach restores function and alleviates pain; follow-up showed relief of low-back pain and radicular leg pain with a significant improvement in quality of life. Notably, despite no direct reduction of the fused slipped segment, fusion at the adjacent level(s) (L3–5 in this case) improved sagittal alignment, suggesting that correction of SI does not rely solely on reducing the slipped segment – adjacent-level decompression and fusion can likewise improve overall spinal function while mitigating operative risk. Indications for this strategy include: imaging-confirmed SF at the index level; symptoms dominated by current/adjacent-level stenosis with nerve-root compression; PI–LL mismatch of moderate magnitude that is correctable via adjacent-level lordosis restoration. Contraindications include: high-grade slip with marked PI–LL mismatch; global malalignment requiring large corrective osteotomy; clear dynamic instability.

For patients similar to the present case, although conservative management is generally first-line – particularly when symptoms are mild – surgery is indicated when nonoperative therapy fails with progressive deterioration. Traditional strategies often prioritize reduction of the slipped segment; however, in the context of SF, direct reduction may entail substantial neural and vascular risk. Our case suggests that, when the index level has undergone SF, targeted nerve-root decompression combined with fusion of the adjacent segment(s) can effectively relieve symptoms while avoiding the major complications associated with conventional reduction procedures.^[14–16]^

This case report provides a relatively conservative yet effective therapeutic strategy; however, it has several limitations: it is a single-patient, uncontrolled case report with potential selection and publication bias; follow-up is limited to 3 months, precluding assessment of long-term outcomes (e.g., fusion rate, adjacent-segment degeneration, implant failure) and complications; and although imaging parameters were obtained using a standardized standing full-length lateral protocol and a predefined measurement workflow, measurer bias may still be present; therefore, 2 independent spine surgeons performed blinded, repeated measurements to mitigate bias, which nevertheless cannot be completely eliminated. Future research should focus on: establishing long-term follow-up to clarify the durability of this surgical strategy; conducting controlled studies to verify its effectiveness; and elucidating the mechanistic relationship between SF and SI. In addition, individualized treatment regimens tailored to different pathophysiological mechanisms will be an important direction in the future management of spinal disorders.

LS with SF and SI is a complex spinal condition that poses substantial clinical challenges. In this case, targeted nerve-root decompression combined with adjacent-level fusion successfully relieved symptoms and improved the spine’s biomechanical balance. Although the slipped segment was not directly reduced, this operative strategy offers a practical and effective option for similar scenarios. Looking ahead, studies should pursue more precise, risk-adapted approaches to further enhance outcomes and reduce complication rates.

## Author contributions

**Conceptualization:** Kaiwei Zhang.

**Data curation:** Kaiwei Zhang.

**Formal analysis:** Kaiwei Zhang.

**Investigation:** Kaiwei Zhang.

**Methodology:** Changliang Ou.

**Project administration:** Xu Gao.

**Resources:** Xu Gao, Haozhu Chen.

**Validation:** Yuhai Yan.

**Writing –original draft:** Yuhai Yan.

**Writing – review & editing:** Yuhai Yan.
